# Association between the risk of hypertension and triglyceride glucose index in Chinese regions: a systematic review and dose-response meta-analysis of a regional update

**DOI:** 10.3389/fcvm.2023.1242035

**Published:** 2023-07-31

**Authors:** An-ran Xu, Qiuyu Jin, Zhisheng Shen, Jiaqi Zhang, Qiang Fu

**Affiliations:** ^1^ The First Clinical Medical College, Heilongjiang University of Chinese Medicine, Harbin, China; ^2^ Department of Gastroenterology, The First Affiliated Hospital of Heilongjiang University of Chinese Medicine, Harbin, China; ^3^ Medical Diagnosis Teaching and Research Room, The College of Basic Medicine of Heilongjiang University of Chinese Medicine, Harbin, China

**Keywords:** TyG index, hypertension, observational study, meta-analysis, dose-response analysis, Chinese region

## Abstract

**Background:**

Triglyceride-glucose (TyG) index has been reported to be associated with various cardiovascular diseases in recent years. However, the conclusive association between the TyG index and hypertension was not established in the last meta-analysis. Furthermore, it remains unclear whether a linear relationship exists between these two variables.

**Methods:**

We conducted a comprehensive search of the CNKI, VIP, WanFang Data, CBM, PubMed, EMbase, Web of Science, and The Cochrane Library databases up until May 10, 2023, to identify relevant studies conducted in China. We used Stata version 17.0 and Rstudio version 4.2.1 to analyze the data and assess the association between the TyG index and the risk of hypertension, as well as the dose-response relationship between these two variables. We will select either a random-effects model or a fixed-effects model based on the results of the heterogeneity tests and report 95% confidence intervals accordingly.

**Results:**

In the end, our analysis encompassed 22 studies involving a total of 668,486 participants, comprising 12 cross-sectional studies and 10 cohort studies. Meta-analysis results showed: Analysis of data from China revealed that an elevated TyG index was associated with a higher risk of developing hypertension, as indicated by an OR/HR of 1.36 [95%CI (1.28–1.45) *I*^2 ^= 69.0% *P* < 0.001]. Through meta-regression analysis of multiple covariates, we found that study type, study region, sample size, database source, and study quality score, the above five variables were able to explain 63.0% of the total heterogeneity. The results of the dose-response Meta-analysis showed: The TyG index has a linear relationship with the risk of developing hypertension, as indicated by non-significant coefficients of higher-order terms in the nonlinear model (*P* > 0.05). The linear trend analysis showed that for every one-unit increase in the TyG index, the risk of developing hypertension increased by 1.5 times [1.532 95%CI (1.294, 1.813) *P* < 0.001]. However, this result is influenced by the number of studies included in the dose-response analysis and requires further corroboration.

**Conclusion:**

In the Chinese region, there was an independent association between TyG index and the risk of developing hypertension, with a linear trend. However, the results of the linear trend need to be corrected by the more number of related studies.

**Systematic Review Registration:**

https://www.crd.york.ac.uk/prospero/display_record.php?ID=CRD42023425836.

## Introduction

The incidence of hypertension varies significantly across the globe, showing substantial differences. In the past 30 years, the prevalence and absolute burden of hypertension have continuously increased in low- and middle-income countries, including China and India—the two countries with the largest populations in the world. Blood pressure and hypertension incidence have been rising or have plateaued at most (1–4). In China, as a representative of developing countries, there is a vast population of middle- and low-income groups. However, economic development and rapid urbanization have led to a rapid transition in the epidemiological and nutritional structure (3, 4). There is research showing that excessive sodium intake is a severe problem in China (5). In addition, some studies have suggested that for residents in rural areas of northern China, especially, changing their unhealthy dietary habits and controlling blood pressure are of immediate importance (6). These changes are also considered the main reasons for the significant increase in cardiovascular disease-related deaths and the continuous rise in the incidence of hypertension in China over the past decade (7, 8). Since the outbreak of COVID-19, hypertension and obesity have been recognized as significant risk factors for exacerbation and complications (9). Meanwhile, in recent years in China, the general public has remained relatively indifferent towards hypertension, despite it being the cardiometabolic risk factor that causes the greatest cardiovascular burden in the country (8). Therefore, early identification of possible hypertension groups, developing primary prevention strategies, and delaying the onset of hypertension is urgent. Diabetes, dyslipidemia, and being overweight or obese are among the most common risk factors for hypertension (10). And these risk factors are closely related to insulin resistance. Insulin resistance can explain the association with hypertension in at least the following ways: Overstimulation of sympathetic excitation (11), Affects the renin-angiotensin-aldosterone system through hyperinsulinemia (12), Significant effects on endothelial structure and function and immune dysfunction (13). And some patients have insulin resistance in prediabetes or hypertension (12), so early identification of the group with insulin resistance is necessary to prevent the development of diabetes and hypertension. Previously, the gold standard for assessing insulin sensitivity was the high insulin-glucose clamp technique. However, this expensive technique is not universally applicable to non-developed areas. To address the issue of cost, the HOMA-IR index was primarily used in the past to assess insulin resistance. However, the recent emergence of the TyG index is expected to replace this index (14). It has been demonstrated that TyG index has a high sensitivity in identifying insulin resistance and its ability to assess insulin resistance and cardiovascular disease more comprehensively and conveniently compared to HOMA-IR index (15). In addition, an increasing number of studies have found that TyG index is closely related to cardiovascular diseases (16). However, the previous research on the association between TyG index and the risk of hypertension did not provide a precise causal relationship, and the number of studies included was relatively small. Additionally, subgroup analysis did not receive an adequate explanation, and there was a lack of research on dose-response (17). Indeed, in the past two years, there have been many studies focusing on the association between TyG index and hypertension in China. This has practical significance to conducting the research for the Chinese region because the changes in hypertension among populations in different regions of the world vary considerably, and study outside of China is relatively scattered. Therefore, we again performed a meta-analysis and conducted a dose-response relationship study to provide a regional answer.

## Methods

Our meta-analysis for this observational study was done under the PRISMA (preferred reporting items for systematic reviews and meta-analyses) statement (18) and MOOSE (meta-analysis of observational studies in epidemiology) statement (19), and its reporting criteria follow the comments of the DRMA reporting guidelines (G-Dose Checklist) provided by Chinese evidence-based medicine (20). The study protocol is registered on the PROSPERO platform (International prospective register of systematic reviews). Registration number: CRD42023425836.

### Search strategy

We searched Chinese and English databases for studies involving the Chinese region separately, and we also referred to the studies covered in the previous meta-analysis (17), which was searched until May 10, 2023. The major database sources used in this study include CNKI, VIP, Wanfang Data, CBM, PubMed, EMbase, Web of Science, and The Cochrane Library. Medical subject terms cannot be searched for Chinese databases, so we mainly rely on comprehensive free terms for our search operations, such as (1) Observational studies, Cross-sectional studies, etc. (2) TyG index, Triacylglycerol-glucose, etc. (3) Hypertension. We utilize English databases and medical subject terms for our search, for example (1) “Hypertension” [MeSH Terms], etc. (2) Triglyceride-glucose [All Fields], etc. (3) “Cross-Sectional Studies” [MeSH Terms], etc. Please refer to Supplementary File S1 for details regarding the search method. There are no restrictions on the language of the study, but the study must take place in China, and the subjects must be “human” and not “animal.” We also retrieved the reference lists of related studies to identify additional relevant articles.

### Inclusion and exclusion criteria

Articles meeting the following criteria will be included in the meta-analysis: Inclusion criteria (1) Study design: Observational studies (Cross-sectional studies, Cohort studies, etc.). (2) Methods and outcomes: Any study reporting associations between the TyG index and hypertension. Exclusion criteria: (1) Studies conducted outside of China; (2) Studies lacking relevant OR, RR, or HR values for necessary outcome reporting; (3) Studies without direct comparison of hypertension and the TyG index; (4) Studies without reporting of any excluded confounding factors; (5) Non-core journal papers published in Chinese databases; (6) The journal where the study is published has been removed from the database; (7) Repeatedly published articles, conference proceedings, and reviews; (8) Special groups, such as pregnant women, children, etc. Two researchers(AR Xu, QY Jin) independently screened the studies and discussed and resolved the controversial ones.

### Data abstraction

Two researchers(AR Xu, QY Jin) independently extracted the study data using the same Excel spreadsheet. Any elements that generated controversy were referred to the third researcher(Q Fu) for discussion and resolution. The content of our subgroup analysis had been predetermined in advance, and thus, our main extracts included: (1) the First author's name and publication year of the study; (2) the specific region in China where the study was conducted; (3) the study design; (4) participant characteristics; (5) mean age (calculated for studies where age was not provided); (6) male to female ratio, along with corresponding effect sizes and intervals (only studies with relevant reports were extracted); (7) sample size. (8) Report format, cutoff value, and effect size with 95% confidence interval for TyG index {for studies that did not provide continuous variables, the effect sizes were obtained by reasonable selection or combination}. (9) Mean BMI {no studies providing mean BMI, obtained by calculation}. (10) Content of adjusted confounders. (11) Relevant data for dose-response analysis.

### Quality assessment

Two investigators (ZS Shen, JQ Zhang) independently evaluated the results of the included studies and the final meta-analysis, in which studies that were too controversial were referred to a third investigator (Q Fu) for discussion and resolution. Only two study types, cross-sectional studies and cohort studies, were ultimately included in this paper. Due to different types of studies requiring different assessment scales, therefore, the cross-sectional study was evaluated with The Agency for Healthcare Research and Quality (AHRQ) scale (21), which consists of 11 evaluation segments and specifies a maximum score of 11. The cohort study was assessed using The Newcastle-Ottawa Quality Assessment Scale (NOS) (22), with a maximum score of 9. There were differences in the total scores of the two scales, and in order to standardize the evaluation criteria for study quality, we subsequently converted the scores of all studies to a total of 10 points. Finally, we evaluated the final evidence quality of the meta-analysis using the GRADE (23) (the Grading of Recommendations Assessment, Development and Evaluation system) rating scale, which is divided into four levels including: high, moderate, low, and very low.

### Statistical analysis

We performed statistical analysis of study data using Stata version 17.0 and Rstudio version 4.2.1. We extracted continuous-type variables, including OR, RR, HR values, and their 95% confidence intervals for all studies after adjusting for confounders. We first combined the effect sizes of all studies and selected either a random effects model or a fixed effects model based on heterogeneity. The combined heterogeneity was assessed using Cochran's Q statistic and *I*^2^ statistic, where *P* < 0.0001 was considered heterogeneous (24). The level of heterogeneity was assessed using *I*^2^ and H statistics, where *I*^2 ^< 50% indicated mild heterogeneity, *I*^2 ^> 75% indicated high heterogeneity (25), H = 1 indicated no heterogeneity, H < 1.2 indicated homogeneity among studies, and H > 1.5 indicated heterogeneity among studies. Galbraith plots were also used to detect heterogeneity. Afterward, we conducted meta-regression analysis and subgroup analysis to explore the between-group differences and sources of heterogeneity. Subgroup analyses were determined *a priori*, including region, study type, age, gender, sample size, database source, mean BMI value, population characteristics, and study quality score. We then used Egger and Begg tests and visual inspection of funnel plots to assess the presence of publication bias (26) and the trim-and-fill method (27) to determine whether the resulting publication bias had an impact on the analysis results. Finally, we used a case-by-case elimination method to determine the stability of the final analysis results. We also extracted studies that were able to enter the dose-response analysis and first performed effect size pooling and exploration of publication bias similar to that described above. Afterward, the lowest dose was used as the control group, while studies that did not provide an exposure dose used the median of the proximal interval as the established dose. The nonlinear model of dose-response was constructed using a three-node restricted cubic spline function (three nodes with positions of 10%, 50%, and 90%) and the generalized least squares estimation method, and the two were tested for association and whether they were linearly related by the Wald method. If *P* < 0.05 indicates that the two are related after passing the regression coefficient of each sample bar of the test. If *P* > 0.05 after testing the coefficients of the higher-order terms in the model, it indicates that the two are linearly related. Finally, we fitted a linear fixed-effect dose-response model and plotted it.

## Results

### Literature search

**Figure 1:**
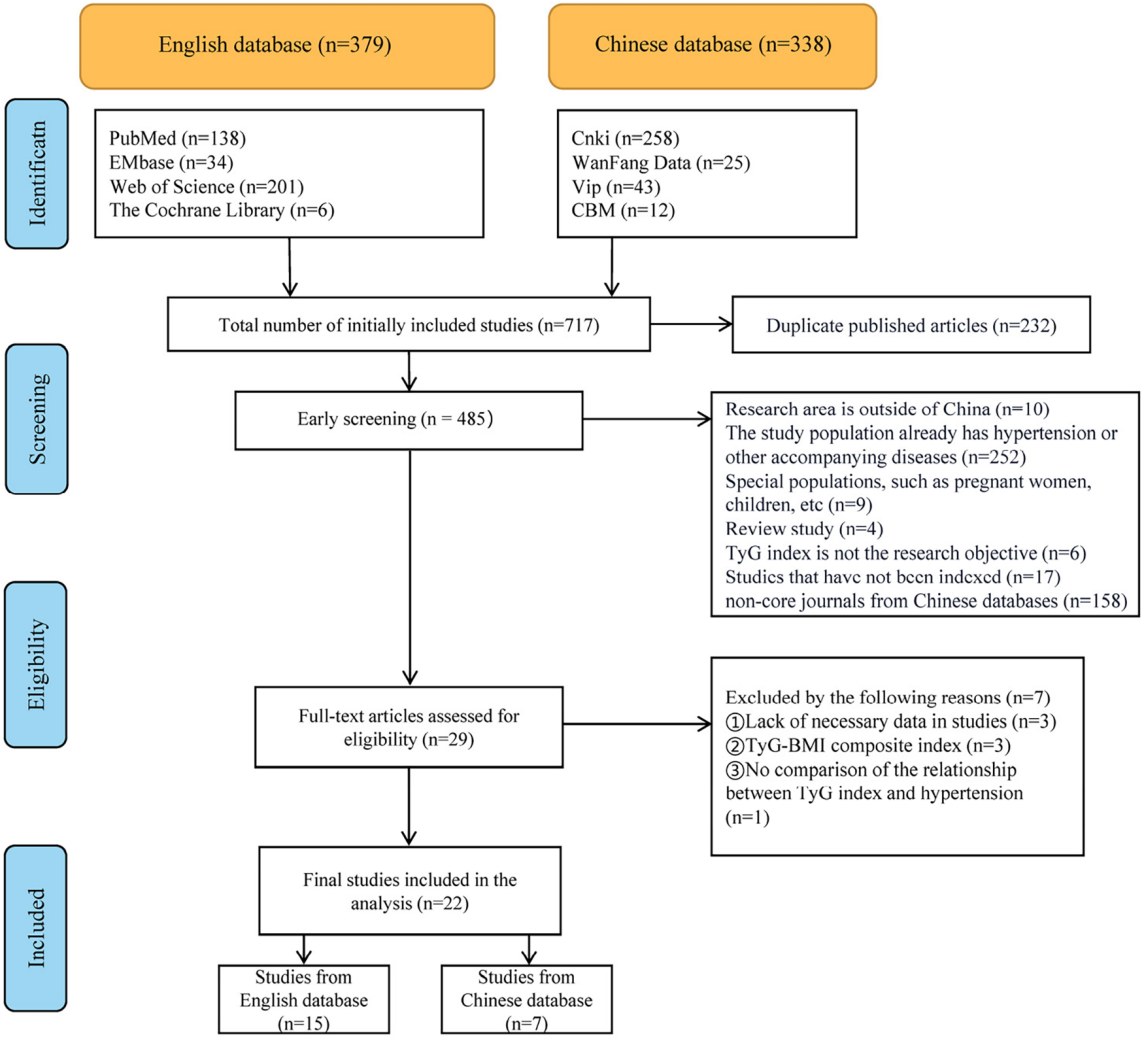
Flow diagram of the literature search and selection.

According to a predetermined search strategy, we obtained a total of 717 pre-screened studies by searching Chinese and English databases. After briefly reading the abstracts, we excluded duplicate publications and articles that did not meet the inclusion criteria. We then read the remaining 29 studies in detail and excluded studies reporting unclear results, incomplete content, or those that did not directly address the association between TyG index and hypertension. Ultimately, 22 studies were included in our meta-analysis, as shown in Figure 1.

### Study characteristics and quality evaluation

Ultimately, we included a total of 22 studies involving 668,486 participants, including 12 cross-sectional studies (28–39) and 10 cohort studies (40–49). The participants were mainly from the general population in urban or rural areas, and a few studies mentioned special occupational groups. All studies declared that they had excluded certain specific diseases (such as diabetes) and did not emphasize whether participants had any specific medical conditions. The mean age of participants ranged from a minimum of 40 years to a maximum of over 60 years, and their mean BMI was generally in the normal range or slightly high. The sample size ranged from 381 to 298,652. The effect sizes and confidence intervals of TyG indices in some of the included studies were reported in the categorical form in some articles. There was a lack of reporting on continuous variables, so we first selected an appropriate effect value as a continuous variable. The requirement for its selection was that it should not be too extreme and needed to be representative while preferring to select a small effect size as a representative. Secondly, if none of the effect sizes of the classifications in the study were representative, they were combined and then used as the continuous-type variable for the study. After conversion, the minimum quality of the articles was 4.55, and the maximum quality was 8.89 on an evidence quality evaluation scale of 10 out of 10, with most of the articles scoring above 7, indicating that the included studies were generally of good quality. Refer to Table 1 and Supplementary File S2 for more information.

**Table 1:** Characteristics of the included observational studies.

Study	Study area	Study design	Characteristics of participants	Mean age (years)	Male (%)	Number of participants	TyG index analysis	Mean BMI index	Variables adjusted	NOS(9)/AHRQ(11) (Converted scores)
Lingfeng Luo 2022	Nationwide	Cross-sectional study	General population middle-aged and elderly people (40–80)	57.78	36.6	5,099	Categorized (None) Continuous 1.10 (0.67–1.70) cutoff -	23.18	Age, Gender, TC, TG, HDL-C, LDL-C, FBG, UA, HBA1c	7 (6.36)
DanYing Deng 2023	Southern China	Cross-sectional study	General population residence time exceeding six months (35–75)	53.70	38.9	92,545	Categorized (Q4:Q1) Continuous 1.49 (1.22–1.82) cutoff -	23.8	Age, Gender, marriage, farmer, annual household income, smoking, drinking, having medical insurance, diabetes, dyslipidemia, history of CVDs, WC, TC, LDL-C, HDL-C	8 (7.27)
Yang Zhao 2023	Northern China	Cohort study	General population rural population	48.00	39.5	10,309	Categorized (Q4:Q1) Continuous 1.30 (1.08–1.57) cutoff -	23.53	Gender, Age, marital status, educational level, monthly income, smoking, alcohol drinking, physical activity, and family history of hypertension, TC, HDL-C	7 (7.78)
Rongjiong Zheng 2017	Southern China	Cohort study	General population (20–80)	40.51	67.8	4,686	Categorized (Q4:Q1) Continuous 1.49 (1.16–1.93) cutoff -	22.46	BMI, WC, BUN, Cr, FPG, UA, AST, ALT, *γ*-GGT, TC, TG, HDL-C, LDL-C, Apo-A1, Apo-B, eGFR	7 (7.78)
Jiwei Zhang 2023	Northern China	Cohort study	General population (18–80)	35.80	25.3	4,347	Categorized (Q5:Q1) Continuous 1.55 (1.18–2.04) cutoff -	20.9	Age, Gender, BMI, ALT, AST, TC, LDL-C, HDL-C	7 (7.78)
Qi Gao 2023	Southern China	Cohort study	General population	48.10	44.7	4,600	Categorized (Q4:Q1) Continuous 1.17 (1.04–1.31) cutoff -	22.9	Gender, Age, BMI, WHR, SBP, DBP, smoking, drinking, region, urban resistance, marital status, education, occupation, dietary intake of fat, protein, carbohydrate, diabetes mellitus, eGFR, BUN, uric acid, hsCRP, hemoglobin, total protein, LDL-C, TC, HbA1c, FBI	8 (8.89)
Yingjie Tian 2023	Northern China	Cross-sectional study	General population permanent resident	55.52	40.2	381	Categorized (Q2:Q1) Continuous 1.88 (1.25–3.11) cutoff 7.02	25.07	Age, SBP, DBP, total cholesterolm, triglycerides, LDL-C, fasting blood glucose	9 (8.18)
Yuanjing Zhu 2022	Southern China	Cohort study	College teachers	49.11	44.9	602	Categorized (Q4:Q1) Continuous 1.55 (1.03–2.33) cutoff -	22.94	Gender, Age, BMI, γ-GGT, ALT, TC, LDL-C, HDL-C, UA, BUN	6 (6.67)
Jing Dong 2022	Northern China	Cohort study	General population employees of schools, companies or local government organizations (>45)	49.42	45.5	4,234	Categorized (None) Continuous 1.39 (1.23–1.57) cutoff -	24.28	Age, Gender, education, smoking, drinking, exercise, obesity, dyslipidemia	8 (8.89)
Binruo Zhu 2020	Nationwide	Cross-sectional study	General population (>40)	57.71	30.1	43,591	Categorized (None) Continuous 1.33 (1.18–1.51) cutoff -	24.24	Age, center, Gender, history of CVDs, history of T2DM, hypoglycemic drugs, SBP, DBP, BMI, ALT, AST, WHR, eGFR, smoking habits, drinking habits	7 (6.36)
Qianyi Xu 2021	Northern China	Cohort study	General population rural areas of Northeast China	52.28	48.9	4,974	Categorized (None) Continuous 1.32 (1.18–1.47) cutoff -	24.36	Age, Gender, Ethnicity	8 (8.89)
Yuqing Li 2023	Nationwide	Cohort study	General population	>45	46.1	4,423	Categorized (None) Continuous 1.63 (1.27–2.11) cutoff male(8.485) female(8.293)	22.89	Age, educational levels, marital status, live place, current smoking, alcohol drinking, activities, exercises, chronic diseases	7 (7.78)
Wenke Cheng 2022	Nationwide	Cross-sectional study	General population	41.00	46.2	117,056	Categorized (Q4:Q1) Continuous 1.11 (1.05–1.16) cutoff 8.19	23.2	Age and Gender	9 (8.18)
Fomin Zhang 2021	Nationwide	Cross-sectional study	General population Permanent resident	51.50	41.2	11,533	Categorized (Q4:Q1) Continuous 1.38 (1.26–1.51) cutoff -	26.3	Age, Gender, smoking, drinking, marital status, and BMI	7 (6.36)
MUHEIYATI Guliman 2023	Northern China	Cross-sectional study	Oilfield workers	43.09	62.4	2,316	Categorized (Q4:Q1) Continuous 1.48 (1.04–2.11) cutoff -	24.27	Age, Gender, smoking, BMI, UA	5 (4.55)
Mingfei Jiang 2022	Southern China	Cross-sectional study	General population	47.08	41.9	298,652	Categorized (Q3:Q1) Continuous 1.56 (1.10–2.21) cutoff 8.85	23.8	Age, Gender, BMI, smoking, drinking, eGFR	8 (7.27)
Song Jian 2017	Southern China	Cross-sectional study	General population residence time exceeding six months (>40)	60.82	42.1	1,777	Categorized (Q4:Q1) Continuous 1.34 (0.94–1.91) cutoff male(9.04) female(8.59)	24.82	Age, BMI, WHR, WHtR, smoking, Gender, family history of hypertension, educational level, marital status and family income	7 (6.36)
Qian Cai 2022	Northern China	Cross-sectional study	General population (18–75)	51.40	38.4	16,793	Categorized (None) Continuous 1.68 (0.95–2.97) cutoff -	24.3	Age, Gender, pulse, TC, TG, FBG, HDL-C, LDL-C, BMI, abdominal obesity, smoking history, binge drinking	7 (6.36)
Wei Luan 2022	Southern China	Cohort study	General population	42.90	45.7	5,504	Categorized (None) Continuous 1.23 (1.04–1.46) cutoff -	22.54	Gender, Age, BMI, ethnicity, smoking status, alcohol consumption, total cholesterol, HDL, LDL, family history of hypertension, excessive salt intake	8 (8.89)
Shiyi Shan 2023	Nationwide	Cross-sectional study	General population (>45)	59.00	47.0	8,209	Categorized (None) Continuous 1.45 (1.33–1.58) cutoff -	None	Age, Gender, residence, education, economic status, tobacco use, alcohol consumption, BMI, WC, HDL-C, LDL-C, high sensitivity C-reactive protein	9 (8.18)
Ruonan Wang 2021	Northern China	Cohort study	General population	43.60	57.7	23,581	Categorized (Q4:Q1) Continuous 1.33 (1.11–1.59) cutoff -	None	Gender, smoking status, alcohol consumption, physical activity, BMI, TC, UA	8 (8.89)
Xin Zhang 2022	Southern China	Cross-sectional study	General population (>18)	40.13	46.9	3,274	Categorized (Q4:Q1) Continuous 1.52 (1.20–1.32) cutoff 8.338	22.2	Age, Gender, BMI, smoking, alcohol, DM	7 (6.36)

TC, Total cholesterol; TG, Triglyceride; HDL-C, High-density lipoprotein cholesterol; LDL-C, Low-density lipoprotein cholesterol; FBG, Fasting Blood Glucose; UA, Uric Acid; HbA1c, Glycated Hemoglobin; BMI, Body Mass Index; WC, Waist Circumference; BUN, Blood Urea Nitrogen; Cr, Creatinine; FPG, Fasting Plasma Glucose; AST, Aspartate Transaminase; ALT, Alanine Aminotransferase; γ-GGT, Gamma-Glutamyl Transferase; Apo-A1, Apolipoprotein A1; Apo-B, Apolipoprotein B; eGFR, Estimated glomerular filtration rate; WHR, Waist-to-Hip Ratio; SBP, Systolic Blood Pressure; DBP, Diastolic Blood Pressure; hsCRP, High Sensitivity C-Reactive Protein; FBI, Fasting blood insulin; CVD, Cardiovascular Disease; T2DM, Type 2 diabetes mellitus; WHtR, Waist-to-Height Ratio; DM, Diabetes Mellitus.

### Meta-analysis results

**Figure 2:**
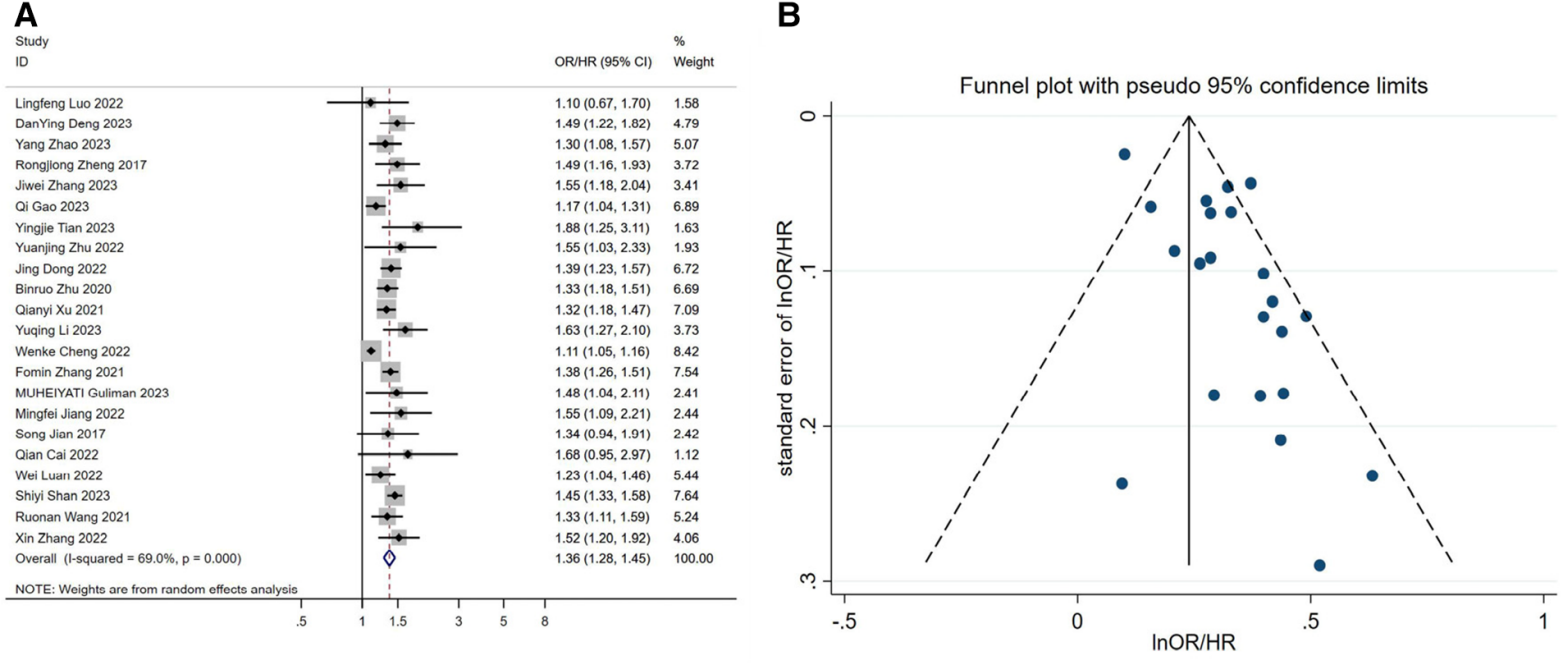
(**A**) forest plot of the association between TyG index and the risk of hypertension. (**B**) Funnel plot of the association between TyG index and the risk of hypertension. OR: Odds Ratio; HR: Hazard Ratio.

After including continuous-type variables from 22 studies and performing a meta-analysis, we found that elevated TyG index increased the risk of developing hypertension, or that this would result in significantly higher blood pressure in patients [OR/HR = 1.36 95%CI (1.28, 1.45), *P* < 0.001, Figure 2A].

**Figure 3:**
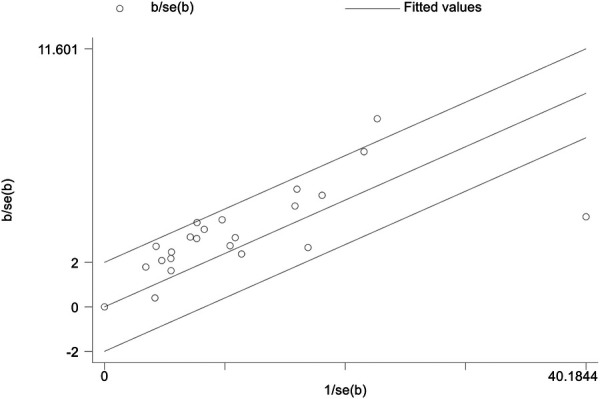
Galbraith plot of the association between TyG index and the risk of hypertension.

In the heterogeneity test results, we found that H = 1.8 > 1.5, *P* < 0.0001, *I*^2 ^= 69.0%, indicating moderate heterogeneity in the meta-analysis (Supplementary File S3). Additionally, when considering the Galbraith plot, we identified a few points falling outside the confidence interval of the regression line, providing further evidence of some level of heterogeneity (Figure 3). In order to identify the source of heterogeneity, we conducted pre-specified subgroup analyses and meta-regression analyses. Through meta-regression analysis, we found that in the regression analysis of individual covariates, study type, study region, mean age, gender, sample size, mean BMI, database source, and study quality score all had an impact on the final results, leading to inter-group differences (*P* < 0.0001). This indicates the need for subgroup analysis. Based on the results of individual covariate regression analysis, we found that study type, study region, sample size, database source, and study quality score may be sources of heterogeneity. Therefore, after including these variables in the multiple covariate regression analysis, we found that the results were statistically significant (*P* = 0.007). The heterogeneity among studies decreased from tau^2 ^= 0.0122 to tau^2 ^= 0.002693, indicating that the introduction of these five variables explains 63.0% of the total heterogeneity. Additionally, *I*^2^ also decreased to 10.63%, indicating that the remaining heterogeneity among different studies, relative to the unexplained variation, is 10.63% after the inclusion of these variables. This suggests that the heterogeneity is associated with these five variables. Finally, no type I error was found in the test using the Monte Carlo permutation method. Please refer to Table 2 and Supplementary File S3 for detailed information.

**Table 2:** Summary of results from the subgroup analyses and regression analysis.

Subgrouped by	No. of studies	OR/HR (95%CI)	*I*^2^ (%)	*P* (overall effect)	*P* (Single covariate)	*P* (Multiple covariates) = 0.007
Study design	22	1.36 (1.28–1.45)	69.00	<0.0001	<0.0001	0.231
Cross-sectional study	12	1.39 (1.25–1.54)	79.50	<0.0001		
Cohort study	10	1.33 (1.25–1.41)	18.30	0.274		
Study area	22	1.36 (1.28–1.45)	69.00	<0.0001	<0.0001	
Southern China	8	1.35 (1.23–1.48)	28.90	0.197		0.133
Northern China	8	1.37 (1.28–1.46)	0.00	0.747	0.731	
Nationwide	6	1.33 (1.16–1.52)	88.90	<0.0001	0.684	0.333
Participants	22	1.36 (1.28–1.45)	69.00	<0.0001	0.069	
General population	20	1.36 (1.27–1.45)	71.30	<0.0001	0.553	
College teachers	1	1.55 (1.03–2.33)	—	—		
Oilfield workers	1	1.48 (1.04–2.11)	—	—	0.887	
Mean age	21	1.35 (1.27–1.44)	68.70	<0.0001	<0.0001	
<45	7	1.34 (1.17–1.52)	71.70	0.002	0.803	
45–50	5	1.31 (1.18–1.45)	33.60	0.197		
>50	9	1.39 (1.33–1.46)	0.00	0.649	0.428	
Gender	20	1.41 (1.29–1.55)	79.80	<0.0001	<0.0001	
Male	10	1.38 (1.22–1.55)	78.00	<0.0001		
Female	10	1.46 (1.26–1.70)	83.10	<0.0001		
Sample size	22	1.36 (1.28–1.45)	69.00	<0.0001	<0.0001	
<4,000	5	1.52 (1.31–1.76)	0.00	0.851	0.137	
4,000–10,000	9	1.35 (1.26–1.45)	44.90	0.069	0.557	0.887
>10,000	8	1.33 (1.19–1.48)	78.40	<0.0001		0.276
Mean BMI index	20	1.36 (1.27–1.45)	66.70	<0.0001	<0.0001	
<22.9	6	1.38 (1.22–1.56)	54.70	0.051		
22.9–23.9	6	1.31 (1.12–1.52)	67.50	0.009	0.382	
>23.9	8	1.37 (1.30–1.44)	0.00	0.851	0.839	
Database	22	1.36 (1.28–1.45)	69.00	<0.0001	<0.0001	0.782
English database	15	1.37 (1.27–1.49)	77.40	<0.0001		
Chinese database	7	1.32 (1.23–1.43)	0.00	0.605		
Scores	22	1.36 (1.28–1.45)	69.00	<0.0001	<0.0001	
<7	8	1.38 (1.29–1.47)	0.00	0.90	0.215	0.363
7–8	6	1.47 (1.33–1.62)	0.00	0.763	0.056	
>8	8	1.30 (1.17–1.43)	83.40	<0.0001		0.101

Table interpretation: In the regression analysis of a single covariate, we stratified the variable. If the number of layers of variables exceeds two, the dummy variable method is needed for regression analysis, and one dummy variable will become the reference and be omitted, resulting in a missing value. If variables can be quantified, such as (0, 1), regression analysis can be performed directly to obtain an overall analysis result, also resulting in the missing values for quantified variables. In regression analysis with multiple covariates, only five types of factors are included and further explain the source of heterogeneity, so other factors are missing values. Similarly, certain included factors will be divided into dummy variables, and one dummy variable will be omitted, resulting in a missing value. (Supplementary File S3 for details).

**Figure 4:**
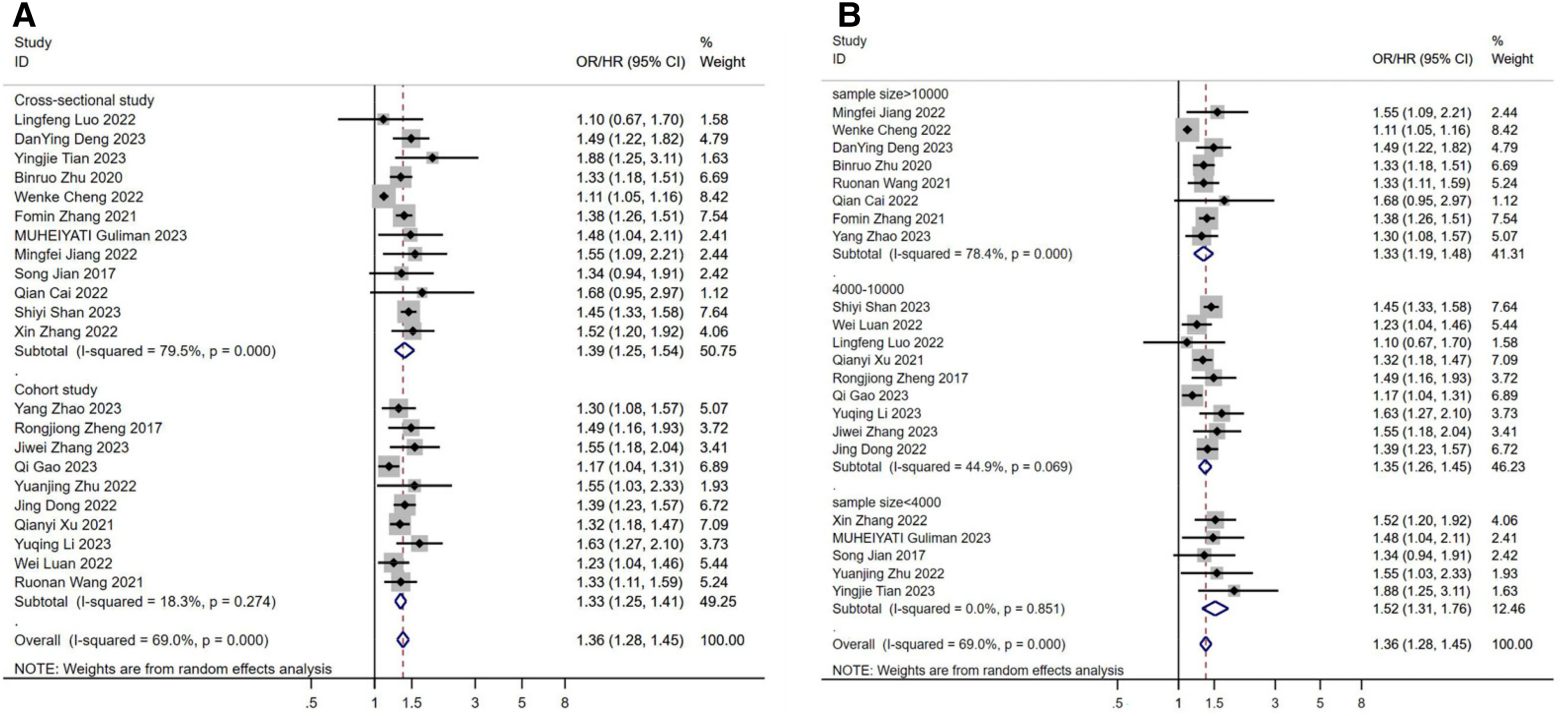
(**A**) subgroup analysis according to the study design. (**B**) subgroup analysis according to the sample size. OR: Odds Ratio; HR: Hazard Ratio.

Study type may be a source of heterogeneity, with significantly lower heterogeneity results in cohort studies and higher heterogeneity results in cross-sectional studies (Figure 4A). Sample size may also be a source of heterogeneity (Figure 4B), and we found that the gradual increase in sample size led to a significant increase in heterogeneity between studies, possibly because the increase in sample size led to a similar increase in confounding factors between individuals. Perhaps after controlling for a certain sample size, the results between studies will stabilize.

We found a relatively large publication bias (Figure 2B and Supplementary File S3) by the funnel plot of visual inspection and Egger's linear regression method with *P* = 0.002. We speculate that the publication bias may stem from the regional nature of the study, as this study was based on the Chinese region. To assess the potential impact of publication bias on the results, we applied the trim-and-fill method to deal with publication bias. Finally, under the fixed-effect model, we found that the estimate before filling was 0.239 [95% CI (0.210, 0.268), *P* < 0.001], and after filling, the estimate was 1.245 [95% CI (1.211, 1.280), *P* < 0.001] (Supplementary File S3). This indicates that although there was a shift in the trim-and-fill results, it was still statistically significant. This suggests that publication bias, although present, has little effect on the meta-analysis results, and the study results remain robust. Ultimately, we performed a sensitivity analysis of the study using the one-by-one exclusion method, which showed little change, and the results of the meta-analysis were stable (Supplementary File S3).

### Results of dose-response relationship analysis

**Figure 5:**
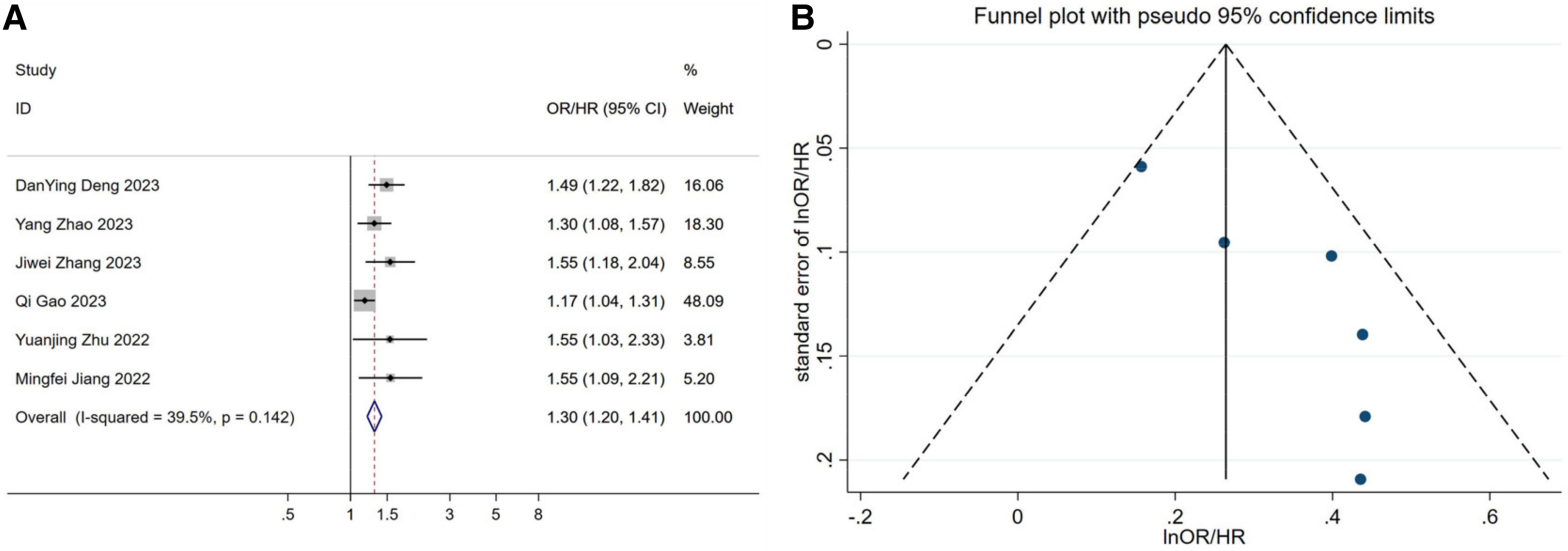
(**A**) forest plot of studies included in the dose-response meta-analysis. (**B**) funnel plot of studies included in the dose-response meta-analysis. OR: Odds Ratio; HR: Hazard Ratio.

**Figure 6:**
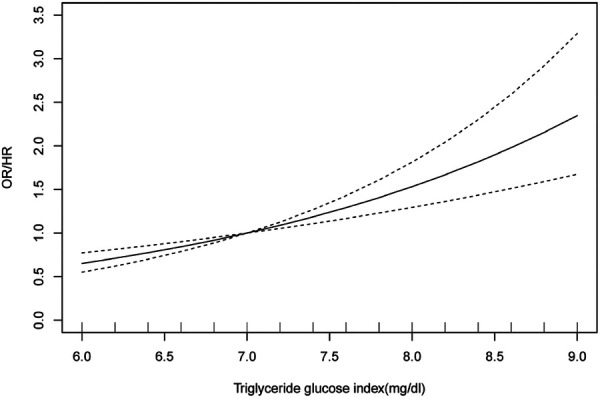
Dose-response analysis plot for the association between TyG index and risk of hypertension. OR: Odds Ratio; HR: Hazard Ratio.

A total of six articles were included in the dose-response analysis (29, 34, 40, 42, 43, 45). After conducting a meta-analysis, the results showed[OR/HR = 1.30 95%CI (1.20, 1.41) *P* = 0.142] (Figure 5A). These findings are generally consistent with the previous analysis results. After fitting a fixed-effects model, the heterogeneity results of the meta-analysis were relatively small (*I*^2^ = 39.5%, *P* = 0.142, H = 1.3). Using the same method, we found that publication bias still existed (Egger's test *P* = 0.021, Figure 5B). After applying the trim-and-fill method based on the fixed-effects model, the before and after results changed but remained statistically significant (Supplementary Figure S3). This indicates that the meta-analysis results are still robust. After the non-linear test of the relationship between TyG index and hypertension using the Wald method to test the coefficient of higher-order variables in the model, the *P*-value was 0.48, which indicates a linear association between the two (Supplementary Figure S3). Therefore, fitting a linear fixed-effect dose-response model yielded the result of 1.532 [95% CI (1.294, 1.813) *P* < 0.001] (Supplementary Figure S3 and Figure 6), indicating that for every one unit increase in TyG index, the risk of developing hypertension increases by 1.5 times.

### GRADE Rating Results

**Figure 7:**
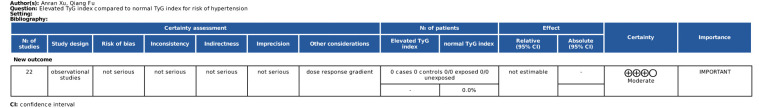
Evaluation of the GRADE grading system for the final meta-analysis results. ⊕, quality of evidence; High quality(⊕⊕⊕⊕), We are very confident that the true effect lies close to that of the estimate of the effect; Moderate quality(⊕⊕⊕), We are moderately confident in the effect estimate: The true effect is likely to be close to the estimate of the effect, but there is a possibility that it is substantially different; Low quality(⊕⊕), Our confidence in the effect estimate is limited: The true effect may be substantially different from the estimate of the effect; **Very low quality(⊕)**, We have very little confidence in the effect estimate: The true effect is likely to be substantially different from the estimate of effect.

The initial quality rating of the results of the observational study meta-analysis was low, but the grade of the results could be upgraded by one level due to the presence of a dose-response relationship, as detailed in Figure 7.

## Discussion

Cardiovascular disease is the leading cause of death worldwide, with most deaths due to coronary heart disease or stroke caused by hypertension, with more than three-quarters of these deaths occurring in low- and middle-income countries (1). China has a large number of low- and middle-income groups, which varies significantly by region. Although the country has spent several years promoting and disseminating primary prevention strategies for hypertension, it seems that the subsequent effects are not satisfactory in the population (7, 8). In China, it is important to be able to easily, conveniently, and quickly identify groups at high risk of developing hypertension. Then the recent emergence of TyG index as an early predictive alarm indicator of hypertension will become valuable. The previous meta-analysis partially reflected the relationship between TyG index and hypertension to some extent. However, the limited number of included studies (5 studies from China, a total of 8 studies) has led to limited interpretation of the results in subgroup analysis and regression analysis. Additionally, the source of heterogeneity was not identified, and no dose-response analysis was conducted. This directly resulted in a lack of sufficient evidence to support a definitive conclusion in the final results (17). In the past two years, the number of relevant articles from China has significantly increased. In comparison, research from other regions of the world is relatively scattered and limited in quantity. For example, studies are distributed in developed regions such as Europe, Japan, and South Korea, as well as developing regions like African countries. However, the average blood pressure and prevalence of elevated blood pressure in high-income regions have significantly decreased. In contrast, blood pressure in East Asia, South Asia, Southeast Asia, Oceania, and Sub-Saharan Africa has shown an upward trend (3, 4). So, studying the relationship between TyG index and hypertension in low- and middle-income populations in East Asia has clear, practical significance. Therefore, this study directly focuses on the population in high-prevalence regions of hypertension (China) to analyze the association between hypertension and TyG index. This approach can provide a more clear and specific result. Also, unlike previous meta-analyses that took values for variables included in the studies, the continuous variables we obtained were basically from the average rather than the highest level of each study, which helped to ensure that the results were not overly magnified.

Based on the results of our observational study's meta-analysis and dose-response analysis, we found a significant and linear association between TyG index and the risk of hypertension or the risk of elevated blood pressure in China [1.532 95%CI (1.294, 1.813) *P* < 0.001]. Regarding the stability of the research results, based on our analysis during the result reporting process, we have found that although publication bias exists, it does not affect the results. Additionally, there was little change in the results after conducting a sensitivity analysis. Although the results of the meta-analysis showed some heterogeneity (H = 1.8 > 1.5, *P* < 0.0001, *I*^2 ^= 69.0%), we later found through regression analysis that the partial heterogeneity was due to the study type, study region, sample size, database source, and study quality score, and these five variables can explain 63.0% of the total heterogeneity.

According to the results of the regression analysis, the study type, study region, mean age, gender, sample size, mean BMI, database source, and study quality score have an impact on the final results, and subgroup analysis is required. In subgroup analysis, the north-south difference in China has a relatively small impact on the results, but its impact on the northern region is slightly higher than that on the southern region, which may be attributed to the fact that blood pressure is generally higher in the population of northern China than in southern China (6). In the subgroup analysis of the average age of the participants, we found that in the older age group, the association between TyG index and hypertension is more pronounced[1.39 95%CI (1.33, 1.46) *P* < 0.001]. In the subgroup analysis of gender, we found that the association between TyG index and hypertension is more pronounced in the female group. This may be because the average age of the participants in this study was around 50 years old, and there is study has shown that the prevalence of hypertension in women before menopause is significantly lower than in men, but after menopause, the prevalence doubles, and is significantly higher than in premenopausal women and age-matched men (50). This may be because after menopause, women's hormones and lipids change, which promotes arterial stiffness and vascular inflammation, leading to high blood pressure (51) while making their relationship with TyG index more closely related. In the subgroup analysis of average BMI, we did not find significant differences between the subgroups, which may be because most of the included studies had already excluded the confounding effects of BMI.

**Figure 8:**
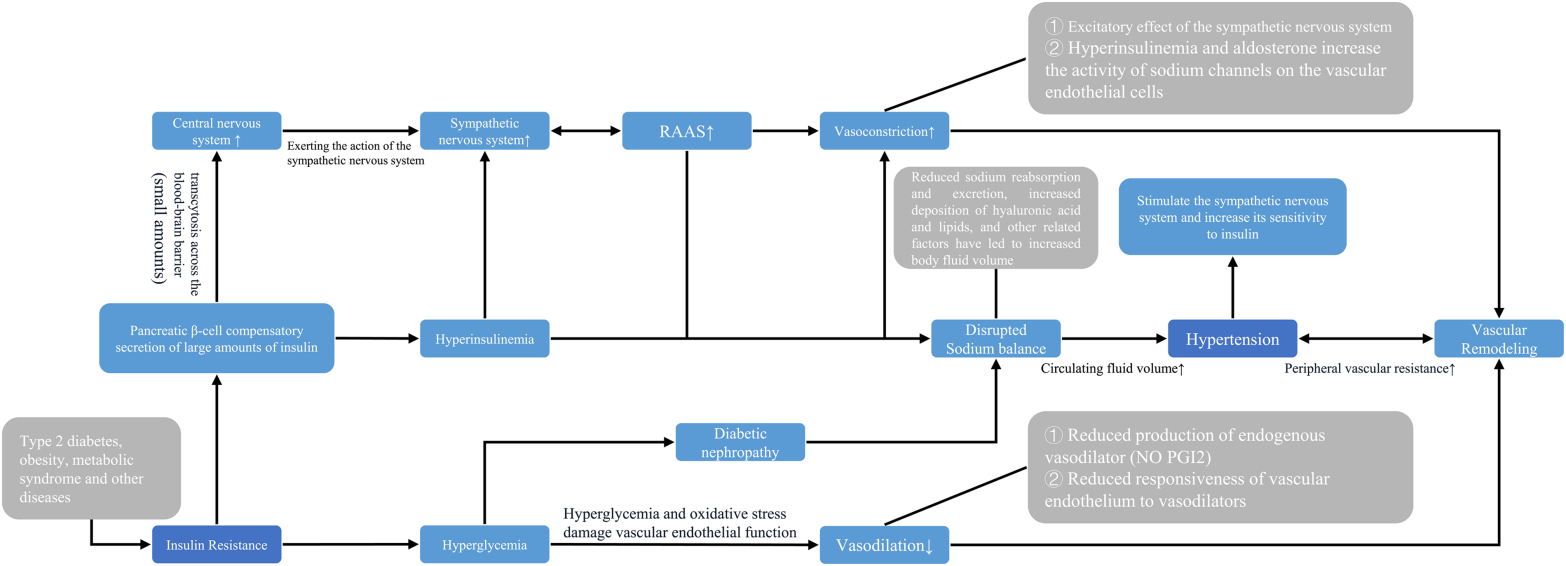
Pathophysiological association between systemic hypertension TyG index. NO: Nitric oxide; PG12 Prostaglandin 12.

In the present study, the ability of the TyG index to predict or monitor hypertension is mainly attributed to its effectiveness in reflecting the degree of insulin resistance. The relationship between insulin resistance and elevated blood pressure can be discussed from the following perspectives: **①** (11–13, 52, 53) The prevailing view in most theoretical studies is that compensatory hypersecretion of insulin by pancreatic Beta-cells in the early stages of diabetes can result in hyperinsulinemia, which can hyper excite sympathetic nerves. It has also been found that during hyperinsulinemia, insulin is able to cross the blood-brain barrier and stimulate central nerves to exert sympathetic excitation. Subsequently, sympathetic excitation increases the secretion of renin, which raises blood pressure by increasing peripheral vascular resistance and cardiac output. Importantly, in the case of concomitant hypertension, the patient will experience further increases in the sympathetic response to insulin, leading to a vicious cycle of continuously increasing blood pressure. **②** (12, 13, 53) Hyperinsulinemia promotes sodium reabsorption by opening the Na^+^/H^+^ transporter expressed in renal tubular cells while also promoting the deposition of intrarenal hyaluronic acid and lipids, which further increases intrarenal pressure. This leads to impaired urine outflow and reduced tubular flow, which in turn increases body fluid volume and drives hypertension. **③** (11, 12) In the middle and late stages of diabetes, insulin resistance still exists, but persistent hyperglycemia and oxidative stress impair vascular endothelial function due to islet β-cell failure. This results in decreased production of endogenous vasodilators and decreased responsiveness of the body to vasodilators, thus reducing the vasodilatory response and promoting a sustained rise in blood pressure. **④** (12, 52) Hyperinsulinemia and aldosterone increase the activity of sodium channels in vascular endothelial cells, while cardiovascular inflammation can impair insulin metabolic signaling and reduce insulin-mediated nitric oxide (NO) production. These physiological changes can lead to arterial stiffness and hypertension (Figure 8).

The TyG index is often associated with insulin resistance or diabetes in predicting hypertension. However, more research is needed to explore other mechanisms that may explain the relationship between the TyG index and hypertension. One advantage of the TyG index over the HOMA-IR index is that it can reflect other factors and their relationship with cardiovascular disease to some extent (54). In addition to insulin resistance and hyperinsulinemia, there are many other factors that are believed to mediate hypertension, including various adipokines from adipose tissue, abnormal states of gut microbiota, and more (54). Although these factors have not been fully confirmed, in several studies, the TyG index has been found to be an independent indicator for describing potential cardiovascular disease (54). Perhaps the re-association of factors such as atherosclerosis, dyslipidemia, and nephropathy with TyG index to explain hypertension or other cardiovascular diseases would be a new option, thus allowing it to be mediated independently of insulin resistance.

However, there are still some limitations in this study. Firstly, the selection or processing of continuous variables included in the meta-analysis may have introduced some bias. Secondly, the results of the regional meta-analysis may not be representative of the association in all regions. Meanwhile, this study still has residual heterogeneity that has not been explained, which means that further in-depth exploration is needed. Finally, the small number of included studies in the dose-response analysis may have an impact on the final linear results.

## Conclusions

Due to the varying and significant differences in the incidence of hypertension worldwide, the hypertension status of populations in developed regions has greatly improved, while the blood pressure situation of populations in middle and low-income areas is not optimistic. Therefore, we conducted this regional discussion. In summary, there is substantial evidence to support the independent and linear association between the TyG index and the risk of hypertension within the middle and low-income populations in China. However, further large-scale clinical studies are needed to precisely determine the numerical value of this linear relationship. Additionally, it is crucial to expand research on the mechanisms underlying the association between the TyG index and hypertension beyond insulin resistance alone.

## Data Availability

The original contributions presented in the study are included in the article/**Supplementary Material**, further inquiries can be directed to the corresponding author.
