# Supplementary material for: Association between the risk of hypertension and triglyceride glucose index in Chinese regions: a systematic review and dose-response meta-analysis of a regional update
Source: Front Cardiovasc Med. 2023 Jul 31;10:1242035. doi: 10.3389/fcvm.2023.1242035 (PMC10424922; doi:10.3389/fcvm.2023.1242035)
Supplement: Supplementary file 1 [file Datasheet1.docx]

**Search strategy**

**Chinese databases**

**CNKI**

**FT= '观察性研究' + '横断面研究' + '队列研究' + '病例对照研究' + '回顾性研究' + '前瞻性研究' + '双向性队列研究' AND SU %= 'Tyg指数' + 'tyg' + '甘油三酯葡萄糖指数' + '甘油三酯血糖指数' + '甘油三酯葡萄糖' + '葡萄糖甘油三酯' + '甘油三酯-葡萄糖' + '三酰甘油葡萄糖' + '三酰甘油-葡萄糖' AND FT = '高血压'**

**Wangfang Data**

**全部:("观察性研究" or "横断面研究" or "队列研究" or "病例对照研究" or "回顾性研究" or "前瞻性研究" or "双向性队列研究") and 全部:("高血压") and 主题:("Tyg指数" or "tyg" or "甘油三酯葡萄糖指数" or "甘油三酯血糖指数" or "甘油三酯葡萄糖" or "葡萄糖甘油三酯" or "甘油三酯-葡萄糖" or "三酰甘油葡萄糖" or "三酰甘油-葡萄糖")**

**VIP**

**M = (Tyg指数 OR tyg OR 甘油三酯葡萄糖指数 OR 甘油三酯血糖指数 OR 甘油三酯葡萄糖 OR 葡萄糖甘油三酯 OR 甘油三酯-葡萄糖 OR 三酰甘油葡萄糖 OR 三酰甘油-葡萄糖) AND U=高血压 AND U=(观察性研究 OR 横断面研究 OR 队列研究 OR 病例对照研究 OR 回顾性研究 OR 前瞻性研究 OR 双向性队列研究)**

**CBM**

**序号 检索表达式 命中文献数 检索时间**

**1) "高血压"[全部字段:智能] 929024 2023-05-13 14:46:41.0**

**2) "观察性研究"[全部字段:智能] OR "横断面研究"[全部字段:智能] OR "队列研究"[常用字段:智能] OR "病例对照研究"[常用字段:智能] OR "回顾性研究"[常用字段:智能] OR "前瞻性研究"[常用字段:智能] OR "双向性队列研究"[常用字段:智能] 3010097 2023-05-13 14:48:24.0**

**3) "Tyg指数"[摘要:智能] OR "tyg"[常用字段:智能] OR "甘油三酯葡萄糖指数"[常用字段:智能] OR "甘油三酯血糖指数"[常用字段:智能] OR "甘油三酯葡萄糖"[常用字段:智能] OR "葡萄糖甘油三酯"[常用字段:智能] OR "甘油三酯-葡萄糖"[常用字段:智能] OR "三酰甘油葡萄糖"[常用字段:智能] OR "三酰甘油-葡萄糖"[常用字段:智能] 979 2023-05-13 14:50:35.0**

**English databases**

**PubMed**

**Search: ((((((cross-sectional study) OR (observational study)) OR (cohort study)) OR (case-control study)) OR (retrospective study)) OR (prospective study)) OR (Ambispective cohort Study) Sort by: Most Recent**

Search: **(Hypertension) OR ("Hypertension"[MeSH Terms])** Sort by: **Most**

Search: (tyg) OR (triglyceride-glucose) Sort by: Most Recent

("tyg"[All Fields] OR "triglyceride-glucose"[All Fields]) AND ("hypertense"[All Fields] OR "Hypertension"[MeSH Terms] OR "Hypertension"[All Fields] OR "hypertension s"[All Fields] OR "hypertensions"[All Fields] OR "hypertensive"[All Fields] OR "hypertensive s"[All Fields] OR "hypertensives"[All Fields] OR "Hypertension"[MeSH Terms]) AND ("cross sectional studies"[MeSH Terms] OR ("cross sectional"[All Fields] AND "studies"[All Fields]) OR "cross sectional studies"[All Fields] OR ("cross"[All Fields] AND "sectional"[All Fields] AND "study"[All Fields]) OR "cross sectional study"[All Fields] OR ("observational study"[Publication Type] OR "observational studies as topic"[MeSH Terms] OR "observational study"[All Fields]) OR ("cohort studies"[MeSH Terms] OR ("cohort"[All Fields] AND "studies"[All Fields]) OR "cohort studies"[All Fields] OR ("cohort"[All Fields] AND "study"[All Fields]) OR "cohort study"[All Fields]) OR ("case control studies"[MeSH Terms] OR ("case control"[All Fields] AND "studies"[All Fields]) OR "case control studies"[All Fields] OR ("case"[All Fields] AND "control"[All Fields] AND "study"[All Fields]) OR "case control study"[All Fields]) OR ("retrospective studies"[MeSH Terms] OR ("retrospective"[All Fields] AND "studies"[All Fields]) OR "retrospective studies"[All Fields] OR ("retrospective"[All Fields] AND "study"[All Fields]) OR "retrospective study"[All Fields]) OR ("prospective studies"[MeSH Terms] OR ("prospective"[All Fields] AND "studies"[All Fields]) OR "prospective studies"[All Fields] OR ("prospective"[All Fields] AND "study"[All Fields]) OR "prospective study"[All Fields]) OR ("Ambispective"[All Fields] AND ("cohort studies"[MeSH Terms] OR ("cohort"[All Fields] AND "studies"[All Fields]) OR "cohort studies"[All Fields] OR ("cohort"[All Fields] AND "study"[All Fields]) OR "cohort study"[All Fields])))

**EMbase**

Session Results

.......................................................

No. Query Results Results Date

#4. #1 AND #2 AND #3 34 13 May 2023

#3. hypertensive 216,520 13 May 2023

#2. 'triglyceride glucose':ti,ab,kw OR 1,576 13 May 2023

triglyceride,glucose:ti,ab,kw OR tyg:ti,ab,kw OR

'triglyceride glucose index':ti,ab,kw OR

'triglyceride-glucose index':ti,ab,kw

#1. 'cross-sectional study'/exp OR 'cross-sectional 4,469,151 13 May 2023

study' OR ('cross sectional' AND ('study'/exp OR

study)) OR 'observational study'/exp OR

'observational study' OR (observational AND

('study'/exp OR study)) OR 'cohort study'/exp OR

'cohort study' OR (('cohort'/exp OR cohort) AND

('study'/exp OR study)) OR 'case-control

study'/exp OR 'case-control study' OR ('case

control' AND ('study'/exp OR study)) OR

'retrospective study'/exp OR 'retrospective

study' OR (retrospective AND ('study'/exp OR

study)) OR 'prospective study'/exp OR

'prospective study' OR (prospective AND

('study'/exp OR study)) OR 'ambispective cohort

study' OR (ambispective AND ('cohort'/exp OR

cohort) AND ('study'/exp OR study))

.......................................................

**Web of Science**

<https://www.webofscience.com/wos/alldb/summary/ab0e7ced-b332-4d5e-af54-a19facd27f14-89afdb0a/relevance/1>


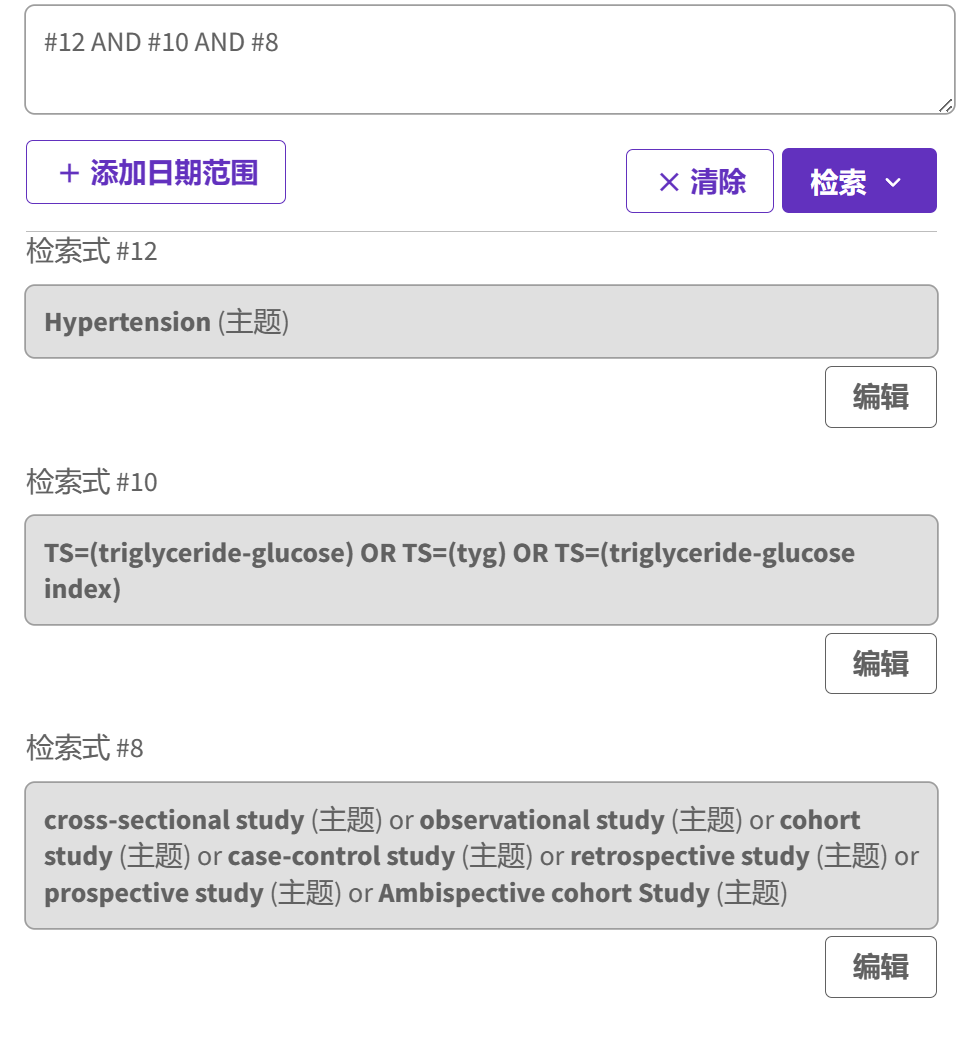


**The Cochrane Library**

Search Name: 123

Date Run: 13/05/2023 07:58:46

Comment:

ID Search Hits

#1 (hypertensive) 19244

#2 (prospective study) OR (observational study):ti,ab,kw OR (cohort study):ti,ab,kw OR (retrospective study):ti,ab,kw OR (cross-sectional study):ti,ab,kw 317576

#3 (triglyceride-glucose):ti,ab,kw OR (tyg):ti,ab,kw OR (triglyceride-glucose index):ti,ab,kw OR (Triglyceride Glucose Index):ti,ab,kw 1685

#4 #3 and #2 and #1 6
